# Early prophylaxis of central venous catheter-related thrombosis using 1% chlorhexidine gluconate and chlorhexidine-gel-impregnated dressings: a retrospective cohort study

**DOI:** 10.1038/s41598-020-72709-w

**Published:** 2020-09-29

**Authors:** Tomoko Yamashita, Ayako Takamori, Akira Nakagawachi, Yoshinori Tanigawa, Yohei Hamada, Yosuke Aoki, Yoshiro Sakaguchi

**Affiliations:** 1 Intensive Care Unit, Saga University Hospital, 5-1-1, Nabeshima, Saga City, Saga 849-8501 Japan; 2 Clinical Research Center, Saga University Hospital, 5-1-1, Nabeshima, Saga City, Saga 849-8501 Japan; 3 Division of Infectious Disease and Hospital Epidemiology, Saga University Hospital, 5-1-1, Nabeshima, Saga City, Saga 849-8501 Japan; 4 Department of Anesthesiology and Critical Care Medicine, Faculty of Medicine, Saga University, 5-1-1, Nabeshima, Saga City, Saga 849-8501 Japan

**Keywords:** Diseases, Medical research, Risk factors

## Abstract

To determine the prophylactic effect of using combined 1% alcoholic chlorhexidine gluconate and chlorhexidine gel-impregnated dressings (CGCD) on catheter-related thrombosis (CRT) in critically ill patients. This retrospective cohort study was performed in an intensive care unit from November 2009 to August 2014. The CRT incidence diagnosed with ultrasound examination was compared between patients applying CGCD and combined 10% aqueous povidone-iodine and standard transparent dressings (PITD) after central venous catheter insertion into the internal jugular vein for ≥ 48 h. CRT was stratified into early (within 7 days) and late (days 8–14) thromboses. Multivariate analyses using logistic regression models clarified the relationships between early- and late-CRT risks and skin antiseptic and catheter site dressing combinations. CRT occurred in 74 of 134 patients (55%), including 52 with early CRT and 22 with late CRT. Patients receiving CGCD had a significantly lower incidence of early CRT than those receiving PITD (odds ratio = 0.18; 95% confidence interval = 0.07–0.45, *p*  < .001). No significant association was evident between using CGCD and late CRT (*p*  = .514). Compared to PITD, CGCD reduced the CRT risk over 7 days in critically ill patients.

UMIN Clinical Trials Registry: UMIN000037492.

## Introduction

Central venous catheters (CVCs) are widely used for therapeutic purposes and for the measurement of hemodynamic variables that cannot be verified from the peripheral veins of critically ill patients. Catheter-related thrombosis (CRT) is a major complication associated with CVCs and a known risk factor for catheter-related bloodstream infections^1^. The reported CRT rates vary widely depending on the study design, patient selection, catheter type and location, and other factors. Previous studies^2–4^ reported CRT rates of 42–56% in the internal jugular veins of critically ill patients, but the efficacy of antithrombotic prophylaxis for CRT remains controversial^5–8^.

The associations between CRT and central line-associated bloodstream infections (CLABSIs) have been described^1,9^. The incidence of bloodstream infections and colonisation was lower in patients with CVC whose skin had been disinfected with > 0.5% alcoholic chlorhexidine gluconate than in patients whose skin had been disinfected with aqueous povidone-iodine^10,11^. Furthermore, using chlorhexidine gluconate-impregnated sponges and chlorhexidine-gel-impregnated dressings reduced catheter-related infection rates in critically ill patients with intravascular catheters^12,13^. However, whether the use of alcoholic chlorhexidine gluconate and chlorhexidine-gel-impregnated dressings may help prevent CRT remains unclear. Therefore, this study aimed to determine the effect of using a combination of 1% alcoholic chlorhexidine gluconate and chlorhexidine-gel-impregnated dressings (CGCD), which are used to prevent CLABSI, on CRT prophylaxis by monitoring CRT for up to 14 days and stratifying CRT according to the time of its occurrence.

## Methods

### Study population

This retrospective cohort study was performed in the intensive care unit (ICU) of Saga University Hospital in Japan. The study protocol adheres to the principles of the Declaration of Helsinki and ethical guidelines for epidemiological research by Japan’s Ministry of Education, Culture, Sports, Science and Technology and Ministry of Health, Labour and Welfare. The protocol was approved by Saga University Hospital’s Ethics Committee (Saga, Japan, Number: 20140402, Date: 20140707), and the need for informed consent was waived given the retrospective nature of the study.

A registry of patients who underwent therapeutic CVC insertions was initiated in November 2009 and was completed in August 2014. To compare CRT incidence among patients administered combinations of antiseptics and dressings (CGCD) or combination of 10% aqueous povidone-iodine and standard transparent dressings (PITD) after CVC insertion, there were included consecutive patients requiring CVC for ≥ 48 h in the ICU, whose CVCs were inserted in the ICU or operating room, and whose catheters were inserted via the internal jugular vein. In contrast, patients with catheters inserted via the subclavian or femoral veins were excluded because a sufficient observation range by ultrasound may not be achieved. Patients aged < 20 years were excluded because CGCD safety and efficacy have not been clarified for young patients. Despite the tendency for the risk of thrombosis to increase with the number of CVC lumens^14^, 12-gauge triple-lumen CVCs were used because most ICU patients required several medications. Patients’ medical care information were verified within their electronic medical charts or similar records. Details of our study were placed on our institution’s homepage, and patients who refused to participate were excluded.

### CVC insertion

The physicians who inserted the CVCs selected 1% alcoholic chlorhexidine gluconate or 10% aqueous povidone-iodine to disinfect the skin before CVC insertion, and the CVCs were inserted percutaneously under ultrasound guidance to minimise catheter insertion trauma, according to a well-established protocol that incorporates maximal barrier precautions. The absence of CRT was verified at the insertion sites using ultrasonography before CVC insertion, and if CRT was present, the patient was excluded. Two types of 12-gauge triple-lumen CVCs were used and provided anticoagulant prophylaxis; these comprised a non-heparin-based biopassive poly (2-methoxyethylacrylate) (PMEA)-coated CVC (CVLEGAFORCE EX; Terumo Corporation, Tokyo, Japan) (type A) and a urokinase and heparin-coated CVC (UA-CV catheter kit; Nihon Covidien Co., Ltd., Tokyo, Japan) (type B). The type of CVC inserted and the CVC exit site, that is, right or left, were at the physician’s discretion. After CVC insertion, chest radiography was used to confirm the position of the catheter tip inside the superior vena cava or innominate vein and to assess whether a pneumothorax was present.

Our clinical approaches towards skin antisepsis and insertion site dressing after CVC insertion changed over time. From November 2009 until December 2010, PITD was used at the insertion sites. The period from January 2011 until August 2012 was excluded from this study because CGCD was phased in to prevent CLABSIs, and this combination was used from September 2012 until August 2014. CGCD was a combination of 1% alcoholic chlorhexidine gluconate (Hexizac AL 1% Cotton Stick 12; Yoshida Pharmaceutical Co., Ltd., Tokyo, Japan) and chlorhexidine-gel-impregnated dressings (3M Tegaderm CHG dressings; 3M Japan Ltd., Tokyo, Japan), and PITD was a combination of 10% aqueous povidone-iodine (Swab Stick Povidone Iodine; LIBATAPE PHARMACEUTICAL Co., Ltd., Kumamoto, Japan) and standard sterile occlusive adhesive transparent dressings (Opsite IV3000; Smith & Nephew KK, Tokyo, Japan). In accordance with Saga University Hospital’s unified ICU manual, nurses prepared and replaced the catheter site dressings immediately if the dressings became damp, loose, or visibly soiled, or as necessary, at least every 7 days.

### Study outcome

The study’s primary outcome was CRT, which was diagnosed using ultrasonography. The internal jugular vein was imaged in the longitudinal and transverse views along its entire length from the angle of the mandible to the supraclavicular fossa^4^. CRT was diagnosed based on the visualisation of an intravascular thrombus, incompressibility of the vein with probe pressure, an absence of spontaneous flow using Doppler techniques^15^, and the requirement for direct visualisation of the thrombus and one or more other signs^4^. An 11-MHz linear probe (Vivid E9; GE Healthcare Japan, Tokyo, Japan) was used. A diagnosis of CRT was established via ultrasound examinations performed routinely at < 12 h before catheter removal. If the catheter was retained for 7 or 14 days, the veins were rechecked for the presence of CRT on days 7 and 14. CRT was monitored for over 14 days. The findings from previous investigations^2,16^ have shown that CRT occurs within 7 days after CVC insertion in > 50% of patients with CRT; therefore, CRT was stratified as follows: CRT within 7 days after CVC insertion was defined as early CRT, and CRT between days 8 and 14 was defined as late CRT.

### Patients’ characteristics

Patients’ characteristics were evaluated, including age, sex, and severity of illness, which were based on the Acute Physiology and Chronic Health Evaluation (APACHE) II score^17^, admission type (medical or surgical), presence of infection and malignancy, administration of prophylactic anticoagulation, CVC exit site (right or left), and CVC type (type A or B).

The patient’s age and sex were obtained on hospital admission. The APACHE II score and admission type were determined by ICU specialists on CVC insertion day. Infections, herein defined as CLABSIs and infections other than CLABSI, were recorded during the CVC insertion period and were diagnosed by infectious disease specialists. CLABSI rates per 1000 catheter-days were recorded. CLABSI was defined according to the Centres for Disease Control and Prevention’s criteria for surveillance of catheter-related bloodstream infection^18^. Hence, patients who met the following criteria were diagnosed with CLABSI: pathogens, excluding common skin contaminants, cultured from one or more blood samples and organisms cultured from blood that were unrelated to infections at any other site, or patients with one or more of the following signs or symptoms: fever (38.0 degrees Celsius [°C]), chills, hypotension, signs and symptoms, and positive laboratory results that were unrelated to an infection at any other site, and a common skin contaminant that was cultured from at least two blood samples drawn on separate occasions. Common skin contaminants were *Corynebacterium striatum*, *Bacillus cereus*, *Bacillus subtilis*, *Propionibacterium acnes*, *Staphylococcus epidermidis*, and *viridans* group streptococci. Malignancy included cancer and haematologic malignancies found until the end of the study period. The attending physicians selected the anticoagulation medicines used and their doses for the prophylaxis of deep vein thrombosis, including CRT, during the CVC insertion period.

### Statistical analyses

Considering the variables describing patients’ characteristics, chi-squared tests or Fisher’s exact test was used to analyse categorical variables, and the Mann–Whitney *U* test was used to analyse continuous variables. Univariate and multivariate analyses were undertaken using logistic regression models to clarify relationships between the risk of early and late CRT and the combination of antiseptic and dressing used.

To determine the risk of early CRT, unadjusted odds ratios (ORs) were estimated without considering patients’ characteristics, and adjusted ORs were estimated using multiple logistic models that included patients’ characteristics as confounders, including age, sex, APACHE II score, admission type, presence of infection and malignancy, prophylactic anticoagulation, CVC exit site, and CVC type. To determine the risk of late CRT, unadjusted ORs were estimated regardless of patients’ characteristics, and adjusted ORs were estimated using multiple logistic models that included patients’ characteristics as confounders, including age, APACHE II score, and CVC type. These confounding variables were selected because they had frequencies of ≥ 10 and stepwise selection had generated the significance levels of 0.2. Then, the combinations of skin antiseptic and catheter site dressing, and age were considered in the stepwise model as variables that should necessarily be included.

The ORs and 95% confidence intervals (CIs) were determined to describe associations. Two-sided values of *p* < .05 indicated statistical significance. The sample size was designed with 58 cases each in the PITD and CGCD groups with a power of 80% and a significance level of 5%. Statistical analyses were performed using JMP version 13.1.0 (SAS Institute Japan, Tokyo, Japan) and EZR version 3.4.1 (Saitama Medical Centre, Jichi Medical University, Saitama, Japan), which is a graphical user interface for R (The R Foundation for Statistical Computing, Vienna, Austria)^19^.

## Results

A total of 1319 patients were treated at Saga University Hospital’s ICU during the study period. Among them, patients were excluded because they remained in the ICU for < 48 h (n = 633), had a CVC inserted outside the ICU or operating room (n = 224), due to the CVC insertion site (n = 136), were younger than 20 years old (n = 19), and due to the catheters selected (n = 173). Finally, we retrospectively analysed 134 patients, among whom 65 received CGCD and 69 received PITD. CRT was present in 74 patients (55%), among whom 52 (39%) had early CRT, and 22 (16%) had late CRT (Table 1).

**Table 1:** Characteristics of patients who did not have or had early- or late-catheter-related thrombosis.

	No CRT^a^ (n = 60)	Early CRT^b^ (n = 52)	*p* value*	Late CRT^c^ (n = 22)	*p* value*
**Skin antiseptic and catheter site dressing combination, n (%)**					
CGCD	39 (65)	14 (27)	< .001	12 (55)	.445
PITD	21 (35)	38 (73)		10 (45)	
Age, years, median,	67	69	.259	71	.946
mean (range)	65 (31–89)	68 (37–92)		62 (26–81)	
**Sex, n (%)**					
Male	35 (58)	33 (63)	.698	18 (82)	.068
Female	25 (42)	19 (37)		4 (18)	
APACHE II score, median,	16	16	.479	15	.156
mean (range)	17 (5–29)	17 (5–38)		15 (7–30)	
**Admission type, n (%)**					
Medical	13 (22)	10 (19)	.817	8 (36)	.253
Surgical	47 (78)	42 (81)		14 (64)	
**Infection, n (%)**	40 (67)	32 (62)	.693	19 (86)	.100
CLABSI, n (%)	1 (1.7)	1 (1.9)	1.000	2 (9.1)	.174
Malignancy, n (%)	16 (27)	16 (31)	.678	5 (23)	.783
Prophylactic anticoagulation, n (%)	30 (50)	28 (54)	.708	18 (82)	.012
**CVC-exit site, n (%)**					
Right	37 (62)	33 (63)	1.000	11 (50)	.449
Left	23 (38)	19 (37)		11 (50)	
**CVC type, n (%)**					
Type A^d^	42 (70)	26 (50)	.035	11 (50)	.120
Type B^e^	18 (30)	26 (50)		11 (50)	

APACHE II, Acute Physiology and Chronic Health Evaluation II; CGCD, 1% alcoholic chlorhexidine gluconate and chlorhexidine-gel-impregnated dressing; CLABSI, central line-associated bloodstream infections; CRT, catheter-related thrombosis; CVC, central venous catheter; PITD, 10% aqueous povidone-iodine and standard transparent dressing.

*The chi-square test or Fisher’s exact test was used to analyse categorical variables, and the Mann–Whitney U test was used to analyse continuous variables.

^a^No catheter-related thrombosis defined as an absence of catheter-related thrombosis during the 14-day period after central venous catheter insertion.

^b^Early catheter-related thrombosis defined as catheter-related thrombosis within 7 days after central venous catheter insertion.

^c^Late catheter-related thrombosis defined as catheter-related thrombosis from day 8 until day 14 after central venous catheter insertion.

^d^Type A was a poly (2-methoxyethylacrylate)-coated central venous catheter.

^e^Type B was a urokinase and heparin-coated central venous catheter.

Table 1 shows the characteristics of the patients with early or late CRT. A significant difference in the skin antiseptic and catheter site dressing combination was evident between patients without CRT and those with early CRT (*p* < .001). There was no significant difference in the skin antiseptic and catheter site dressing combination used between patients without CRT and those with late CRT (*p* = .445).

Table 2 presents the ORs, 95% CIs, and *p* values determined from univariate and multivariate logistic regression models that evaluated associations between early CRT and CGCD use. The univariate analysis showed significant differences in the use of CGCD (unadjusted OR = 0.20; *p* < .001) and type A CVC (unadjusted OR = 0.43; *p* = .032) between patients without CRT and patients with early CRT. The multivariate analysis showed a significant association between CGCD use and a lower incidence of early CRT (adjusted OR = 0.18; 95% CI 0.07–0.45; *p* < .001). Moreover, no significant difference in type A CVC was found between patients without CRT and patients with early CRT (*p* = .185).

**Table 2:** Univariate and multivariate logistic regression analyses of the risks of early CRT with CGCD.

	Early CRT				
	Unadjusted OR	*p* value	Adjusted OR	95% CI	*p* value
CGCD	0.20	< .001	0.18	0.07–0.45	< .001
Age	1.02	.227	1.02	0.98–1.06	.282
Sex (female)	0.81	.580	0.74	0.31–1.76	.491
APACHE II score	0.99	.872	1.00	0.92–1.08	.978
Admission type (medical)	0.86	.750	0.59	0.20–1.76	.343
Infection	0.80	.572	0.75	0.30–1.88	.535
Malignancy	1.22	.632	1.56	0.59–4.12	.372
Prophylactic anticoagulation	1.17	.685	0.96	0.41–2.25	.918
CVC-exit site (left)	0.93	.845	0.76	0.31–1.85	.542
CVC type (type A^a^)	0.43	.032	0.54	0.22–1.34	.185

APACHE II, Acute Physiology and Chronic Health Evaluation II; CGCD, 1% alcoholic chlorhexidine gluconate and chlorhexidine-gel-impregnated dressing; CI, confidence interval; CRT, catheter-related thrombosis; CVC, central venous catheter; OR, odds ratio.

^a^Type A was a poly (2-methoxyethylacrylate)-coated central venous catheter.

Table 3 presents the ORs, 95% CIs, and *p* values determined from univariate and multivariate logistic regression models that evaluated associations between late CRT and CGCD use. The univariate analysis revealed significant differences in prophylactic anticoagulation between patients without CRT and patients with late CRT (unadjusted OR = 4.50; *p* = .014). The multivariate analysis showed that late CRT was not associated with CGCD use (*p* = .514), age, APACHE II score, and CVC type.

**Table 3:** Univariate and multivariate logistic regression analyses of the risks of late CRT with CGCD.

	Late CRT				
	Unadjusted OR	*p* value	Adjusted OR	95% CI	*p* value
CGCD	0.65	.389	0.71	0.25–1.99	.514
Age	0.99	.442	0.99	0.96–1.03	.651
Sex (female)	0.31	.056	–	–	–
APACHE II score	0.95	.248	0.94	0.85–1.04	.216
Admission type (medical)	2.07	.181	–	–	–
Infection	3.17	.090	–	–	–
Malignancy	0.81	.718	–	–	–
Prophylactic anticoagulation	4.50	.014	–	–	–
CVC-exit site (left)	1.61	.344	–	–	–
CVC type (type A^a^)	0.43	.097	0.40	0.14–1.13	.083

APACHE II, Acute Physiology and Chronic Health Evaluation II; CGCD, 1% alcoholic chlorhexidine gluconate and chlorhexidine-gel-impregnated dressing; CI, confidence interval; CRT, catheter-related thrombosis; CVC, central venous catheter; OR, odds ratio.

^a^Type A was a poly (2-methoxyethylacrylate)-coated central venous catheter.

CLABSIs developed in four patients, and the CLABSI rate per 1000 catheter-days was 4.67 during this study. The CLABSI rates were 1.5% (1/65) among patients who received CGCD and 4.3% (3/69) among patients who received PITD; the groups did not differ in the incidence of CLABSI (*p* = .620). The CLABSI rates per 1000 catheter-days were 2.18 for patients who received CGCD and 7.56 for patients who received PITD. Three patients had CRT and CLABSIs.

## Discussion

This study showed that CGCD effectively reduced the incidence of CRT within 7 days compared with PITD. The pathogenesis of CRT is complex and poorly understood; CRT is composed of cellular elements, such as smooth muscle cells, endothelial cells, and noncellular elements, such as fibrin and fibronectin^20–22^. The reason for the decrease in CRT in the CGCD group in this study may be due to the antibacterial and antithrombotic effects of chlorhexidine. Colonization of the catheter occurs predominantly from the skin puncture site during short-term CVC (< 20 days), and microorganisms from the cutaneous microflora invade CVCs extraluminally and form biofilm^23–25^. Chlorhexidine prevents microorganisms’ invasion of the CVC insertion site. Consequently, chlorhexidine inhibits microorganisms and matrix produced by microorganisms from adhering to the catheter. It is expected that the inhibition of this adhesion will reduce thrombus around the catheter. Further chlorhexidine minimizes thrombus accumulation on the catheter surface by the inhibition of thrombin^22^. Thus, CRT formation is promoted by the vicious cycle of thrombus and infection, and the results of this study suggest that chlorhexidine may stop this vicious cycle. In a previous study, a chlorhexidine-coated catheter significantly reduced fibrin sheath formation around the catheter^22^. The fibrin sheath enhances catheter colonization and catheter-related infection^26^. Although the number of patients with CLABSIs was very small in this study, which may account for the insignificant difference in the rate of CLABSI between the CGCD and PITD groups, chlorhexidine reduces the risk of CLABSIs associated with short-term CVC and may prevent CRT. Conversely, this study showed that compared with PITD, CGCD did not reduce the incidence of CRT from day 8 until day 14. The findings of the present study may have been affected by the limited number of patients with late CRT. Thus, additional studies are required to confirm our results.

Moreover, the results of this study could not demonstrate whether the 1% alcoholic chlorhexidine gluconate or the chlorhexidine-impregnated dressings were more strongly associated with CRT prevention compared with PITD on early CRT. We hypothesised that using 1% alcoholic chlorhexidine gluconate would reduce CRT based on the results from our unpublished pilot study (data not shown). However, given that the chlorhexidine-gel-impregnated dressing is highly adhesive, we speculated that it can effectively provide continuous disinfection via chlorhexidine and prevent vessel damage associated with catheter movements that occur when patients move their necks. Previous research shows the mobility of CVCs may be associated with the development of CVC-related complications, such as catheter-related infections and thrombosis^27^. When the catheter is placed inside the vessel, turbulent conditions occur, which lowers the shear rates in the catheter-indwelling portion. Additionally, this promotes the adhesion of platelets and neutrophils to the surface of the fibrinogen and collagen attached to the catheter and vein walls^20,28^. Poor catheter fixation may further impair shear rates and produces endothelial erosions at the insertion site. Thus, poor catheter fixation may result in thrombosis, and the chlorhexidine-gel-impregnated dressing may prevent it. Additional studies should be conducted to confirm our speculation.

The multivariate analysis of early CRT involved adjustment of the ORs using 10 patient characteristics as confounders, regardless of their p values, because these factors were either associated with CRT in previous studies or we anticipated that these factors were important for CRT. We evaluated the risk factors for CRT in this study. The risk of early CRT did not differ according to the administration of prophylactic anticoagulants, and most patients with late CRT were administered prophylactic anticoagulants. We speculated that the results were affected by differences among the prophylactic anticoagulants used and their doses. The efficacy of antithrombotic prophylaxis for CRT is controversial^5–8,29^, and no clear benefit has been determined for critically ill patients who received anticoagulant prophylaxis for CRT. Hence, individualised risk and benefit evaluations are required.

Considering the type of CVC, the multivariate analysis of late CRT showed that type A tended to be associated with a lower incidence of CRT (*p* = .083). Type A comprises a PMEA-coated CVC that is used for pump oxygenators, and it has a high level of biocompatibility^30^. The PEMA-coated catheter prevented the attachment of fibrin sheath and thrombus to the catheter^22,31^. This result on the PEMA-coated catheter in this study may have been affected by the number of patients with late CRT. Hence, additional studies are needed to confirm this finding.

This study has several limitations that should be acknowledged. First, the same antiseptic was not used to clean the skin before CVC for the CGCD group; however, each group (CGCD or PITD) had the same antiseptic used after CVC insertion for that group’s participants. In the PITD group, 10% aqueous povidone-iodine was used for skin disinfection before CVC insertion. In the CGCD group, some patients’ skin was cleaned using 10% aqueous povidone-iodine before CVC insertion based on the physicians’ discretion, and CGCD was used after CVC insertion. This may be an important factor in the study, and results may be better if the same pre-CVC antiseptic solution was used after CVC insertion. Second, we may have underestimated the actual incidence of CRT because we were unable to check for the presence of CRT around the catheters’ distal ends using ultrasonography. However, several studies have evaluated and compared the diagnostic accuracies of ultrasonography and venography for upper extremity thrombosis, and the sensitivities and specificities of ultrasonography for the diagnosis of upper extremity deep vein thrombosis range from 84 to 97% and 88 to 96%, respectively^15,32^. Third, this study involved a small sample size and a limited observation period; thus, further prospective studies are needed to verify the presented results.

## Conclusions

The results of this retrospective study suggested that the use of CGCD reduced the risk of CRT over 7 days in patients in the ICU compared with PITD. Although CRT is not directly life-threatening for critically ill patients, CRT is associated with CLABSI and pulmonary embolism risk; thus, it is better to remove CVC immediately when no longer needed.

## Data Availability

The datasets used and/or analysed during the current study are available from the corresponding author on reasonable request.
